# Association between Risk Communication Format and Perceived Risk of Adverse Events after COVID-19 Vaccination among US Adults

**DOI:** 10.3390/healthcare11030380

**Published:** 2023-01-29

**Authors:** Joshua E. Rosen, Sylvia Seo Eun Chang, Spencer Williams, Joy S. Lee, DaHee Han, Nidhi Agrawal, Joseph H. Joo, Gary Hsieh, Katharina Reinecke, Joshua M. Liao

**Affiliations:** 1 Surgical Outcomes Research Center, Department of Surgery, University of Washington, Seattle, WA 98195, USA; 2 Foster School of Business, University of Washington, Seattle, WA 98195, USA; 3 Department of Human Centered Design & Engineering, University of Washington, Seattle, WA 98195, USA; 4 Department of Medicine, University of Washington, Seattle, WA 98195, USA; 5 Value and Systems Science Lab, Seattle, WA 98195, USA; 6 Desautels Faculty of Management, McGill University, Montreal, QC H3A 1G5, Canada; 7 Paul G. Allen School of Computer Science & Engineering, University of Washington, Seattle, WA 98195, USA

**Keywords:** COVID-19, vaccines, risk communication, risk perception

## Abstract

The format used to communicate probability—verbal versus numerical descriptors—can impact risk perceptions and behaviors. This issue is salient for the Coronavirus disease 2019 (COVID-19), where concerns about vaccine-related risks may reduce uptake and verbal descriptors have been widely used by public health, news organizations and on social media, to convey risk. Because the effect of risk-communication format on perceived COVID-19 vaccine-related risks remains unknown, we conducted an online randomized survey among 939 US adults. Participants were given risk information, using verbal or numerical descriptors and were asked to report their perceived risk of experiencing headache, fever, fatigue or myocarditis from COVID-19 vaccine. Associations between risk communication format and perceived risk were assessed using multivariable regression. Compared to numerical estimates, verbal descriptors were associated with higher perceived risk of headache (β = 5.0 percentage points, 95% CI = 2.0–8.1), fever (β = 27 percentage points, 95% CI = 23–30), fatigue (β = 4.9 percentage points, 95% = CI 1.8–8.0) and myocarditis (β = 4.6 percentage points, 95% CI = 2.1–7.2), as well as greater variability in risk perceptions. Social media influence was associated with differences in risk perceptions for myocarditis, but not side effects. Verbal descriptors may lead to greater, more inaccurate and variable vaccine-related risk perceptions compared to numerical descriptors.

## 1. Introduction

Three years ago, the Center for Disease Control confirmed the first case of Coronavirus disease 2019 (COVID-19) in the United States and scientists believe the virus will not be irradicated for years to come. Vaccines remain as one of the safest strategies to prevent severe disease [1]. However, fear of COVID-19 vaccine-related side effects and adverse events may impede vaccine uptake. Recent studies have shown that many adults rank a fear of COVID vaccine side effects highly in the context of major global health threats [2]. Currently, these risks are described using verbal terms (e.g., common, rare) by major public health as well as media organizations [1]. While well-intentioned, this approach may be problematic because the format for communicating risk—using verbal versus numerical descriptors of probability—can impact risk perceptions and decision making [3,4]. These dynamics are particularly relevant in the age of social media, given its rapid and significant influence on the public’s beliefs, attitudes and behaviors. COVID-19 vaccine information has been widely conveyed via social media platforms [5], where high rates of disinformation may lead to vaccine hesitancy [6].

Verbal descriptors can be associated with inaccurate and inflated perceptions of risk, which in turn affect medical treatment decisions such as choosing to initiate a new drug or enter a clinical trial [3,7,8]. The impact of risk communication methods on risk perceptions can vary by context and condition. Currently, little is known about how risk communication formats affect the perceived risk of both minor and serious adverse events after COVID-19 vaccination.

We addressed this evidence gap by testing how risk communication format—numerical versus verbal descriptors of risk—for three side effects (headache, fever and fatigue) and adverse event (myocarditis) affect individuals’ perceptions of the risk of these events occurring after vaccination against COVID-19. The aim of this study was to assess the impact of risk communication format on risk perceptions. We hypothesized that risk communication format would affect risk perceptions, with verbal descriptors leading to more variable and inflated risk perceptions. We also hypothesized that participants who rate social media as an important source of information would have greater perceptions of vaccine-related risks regardless of the risk communication format.

## 2. Methods

We conducted a web-based randomized survey in January 2022 among adults in the United States who had not received all main series and booster vaccinations. Participants were recruited as a convenience sample using Amazon’s Mechanical Turk (MTurk) crowdsourcing platform, which has been extensively utilized to study healthcare decision making and risk perceptions in US adults [4,9,10]. Eligible participants were ≥18 years old, lived in the United States and had an MTurk approval rating of >98%. We excluded participants who indicated their age > 90. Participants who stated they had not received all main series and booster vaccinations were randomized using 1:1 simple randomization to receive information about headache, fatigue, fever and myocarditis after COVID-19 vaccination using either verbal descriptors or numerical point estimates. Headache, fatigue and fever were described as “common” or 41%, 47%, 8%, respectively, while myocarditis was described as “rare” or “0.001205%” based on data obtained from the US Centers for Disease Control and Prevention website in October 2021 [1]. After receiving information in verbal versus numerical form, participants then rated their perceived risk on a scale of 0–100% in response to the following question: “What is your risk of getting [side/adverse effect] after the [next indicated dose]?”

The survey also captured information about participant demographics and political identity (7-point scale from 0 = Conservative to 7 = Liberal). Participants were asked their intention toward getting the next indicated vaccine dose (definitely, not sure, definitely not); those that reported being unsure were also asked whether they needed more time to decide. Based on these responses, participants were defined as either vaccine accepting (definitely would get next vaccine dose) vaccine rejecting (definitely would not), vaccine deferring (not sure, need more time to decide), or otherwise vaccine hesitant (not sure, do not need more time to decide).

The survey also asked participants to report the extent to which they were impacted by different sources of information about COVID vaccines (7-point scale): social media, loved ones, doctor or other healthcare professional, religious community, work or school community, news media, public health organizations and others in their lives. Participants reporting an impact from social media that was greater than or equal to all other sources were defined as high social media influence.

### Statistical Analysis

Findings were described using means and standard deviations (SD) for headache, fever and fatigue. Given the highly skewed distribution of responses for myocarditis, we described the data using medians and interquartile ranges (IQRs). For each of the four adverse events, a linear regression model with robust standard errors was used to assess the impact of verbal versus numerical risk communication format on individuals’ perceived risk, accounting for sociodemographic characteristics, political identity, vaccine hesitancy and social media influence. All hypothesis tests were two-sided and an alpha of 0.05 was used for statistical significance. Analyses were performed in R version 4.1.0 packages gtsummary, tidyverse and ggplot2 [11].

This study was approved by the University of Washington Institutional Review Board (STUDY00014136). We followed the American Association for Public Opinion Research Reporting guidelines for survey studies (Table S1).

## 3. Results

Overall, 939 participants were included in the analysis; 575 (61%) of whom were fully vaccinated and not boosted, 61 (6.5%) of whom were partially vaccinated and 303 (32%) of whom were unvaccinated. A total of 495 individuals were randomized to numerical descriptors of probability and 444 were randomized to verbal descriptors. Mean age was 39 years and 43% identified as female. Participant characteristics were well balanced between the two study arms (Table 1).

### 3.1. Headache, Fever and Fatigue

**Figure 1:**
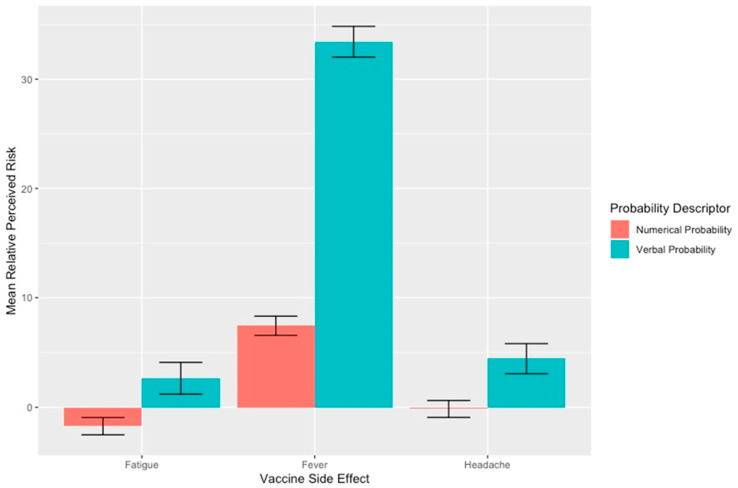
Mean relative perceived risk of side effects from COVID-19 vaccines when communicated using verbal or numerical probability descriptions. Bars show the mean perceived risk of participants—the stated numerical probability so larger bars represent larger deviations from the true risk of the side effect. Error bars represent standard deviations.

**Figure 2:**
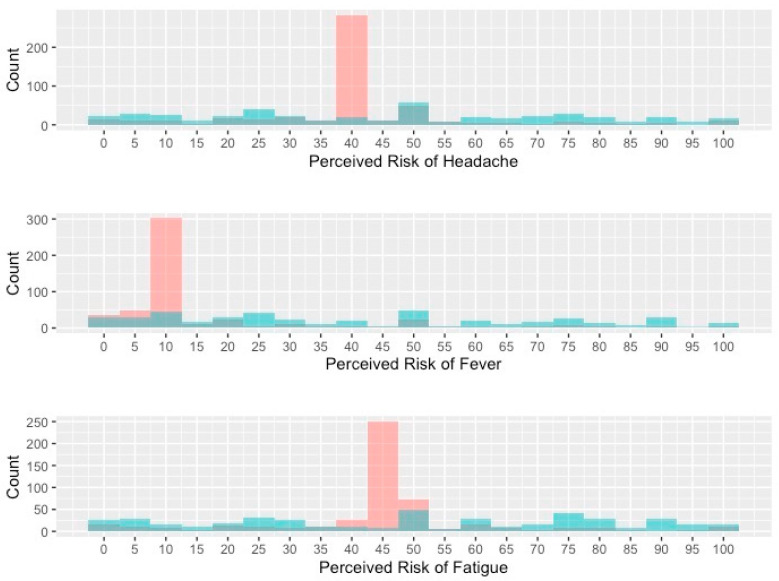
Histograms of participants perceived risk for each complication demonstrating increased variability in risk estimates with verbal probability descriptors. Red bars represent numerical probability descriptors and green bars represent verbal probability descriptors.

For all three “common” side effects, mean risk perceptions were higher and more variable in the verbal descriptor than numerical descriptor arm. These differences were largest for fever, with mean estimated risk in the verbal descriptor of 41.4% (SD = 28.9%) versus 15.4% (SD = 15.4%) in the numerical descriptor arm. Differences between arms were smaller for headache (verbal descriptor mean = 45.4%; SD = 28.9% vs. numerical descriptor mean = 40.9%; SD = 17.2%) and fatigue (verbal descriptor mean = 49.6%; SD = 30.4% vs. numerical descriptor mean = 45.3%; SD = 17.5%). Verbal descriptors also led to greater differences between participants’ risk estimates and published risk estimates (Figure 1), corresponding to greater inaccuracy in risk perceptions. For each side effect, standard deviations were also larger in the verbal versus numerical descriptor arm, indicating increased variability in participants risk perceptions when presented with verbal descriptors of risk (Figure 2).

In multivariable analysis, verbal descriptors were associated with higher perceived risk for all three side-effects: headache (β = 5.0 percentage points, 95% CI = 2.0–8.1), fever (β = 27 percentage points, 95% CI = 23–30) and fatigue (β = 4.9 percentage points, 95% CI = 1.8–8.0) (Table 2).

We did not observe an association between high social media influence and differences in side effect risk perceptions (Table 2). Self-reported political identity was not linearly associated with perceived risk of headache, fever, or fatigue (Table 2). Individuals in the vaccine-rejecting or vaccine-deferring groups were more likely to possess increased perceptions of the risk of headache and fatigue, but not fever compared to individuals in the vaccine-accepting group. In contrast, we did not find increased risk perceptions amongst those categorized as vaccine-hesitant (i.e., those who stated they need more time to decide). Male gender identity was associated with lower mean risk perceptions for all three side effects (Table 2).

### 3.2. Myocarditis

Participants exposed to a verbal descriptor (“rare”) had higher median perceived myocarditis risks and greater variability in perceived risk than those exposed to numerical probabilities (median = 2.0%, IQR 0.1% to 15% vs. median = 0.001%, IQR 0.001% to 1.0%). In multivariable analysis (Table 2), verbal descriptors were associated with a higher perceived myocarditis risk (β = 4.6 percentage points, 95% CI = 2.1–7.2). Participants most influenced by social media also had higher perceptions of myocarditis risk (Table 2). More liberal political identity was associated with a lower perceived risk of myocarditis after vaccination.

Compared to those who were vaccine-accepting, respondents who were vaccine-deferring or vaccine-hesitant expressed lower risk perceptions, with risk perception confidence intervals that included more negative value (though confidence intervals for vaccine-hesitant individuals included small positive values). Vaccine-rejecting individuals did not have a clear difference in risk perceptions compared to those who were vaccine-accepting. Male gender identity was also associated with lower mean risk perceptions of myocarditis (Table 2).

## 4. Discussion

In this randomized survey, verbal descriptors of the COVID-19 vaccine side effect risks led to less accurate, more variable and heightened risk perceptions compared to numerical risk descriptors for several vaccine-related side effects and adverse events.

The main implication of these findings is that going forward, public health, medical and news media organizations may benefit from reconsidering their approach to engaging the public about vaccine-related risks. In particular, our results underscore the potential value of moving away from conveying risks solely using verbal descriptors and providing numerical estimates of risk.

In some circumstances, such changes may require concerted efforts to avoid verbal descriptors. Verbal probability descriptors are attractive and widely used in health and public health communication because they are concise, easy to communicate conversationally and conveyable to individuals with variable numeracy [3,7,12]. However, our findings add to a body of knowledge outside the COVID-19 context and demonstrate that despite good intentions, there are potential pitfalls in using these descriptors [3,4,7,8].

Verbal descriptors permit ambiguity, require interpretation and rely on individuals’ existing subjective sense of risk [3,7,8,12,13]. For all three side effects assessed in this study, participants in the verbal descriptor arm estimated risks consistently between 40 and 50%, likely reflecting internal reference points and connections between the word “common” and that risk range. Because the published risks of developing fatigue and headache also happened to be in this range, there were only small differences between participants’ estimated risk perceptions and published risk estimates. The larger difference of 26% for fever may suggest discrepancies that can arise between participants’ internal reference points and published risk values [8].

Our data regarding myocarditis are particularly striking: median risk perceptions for verbal descriptors were over 1000 times higher than for numerical descriptors. Prior work has shown that inflated risk perceptions caused by verbal descriptors can decrease patient’s willingness to participate in clinical trials [7] or start medications [8]. It is therefore likely that misperceptions of this magnitude may also affect patient’s willingness to undergo vaccination against COVID-19.

Even when the average risk perceptions were similar between numerical and verbal descriptors, our data demonstrate that verbal descriptors had much higher variability in risk perceptions at the individual level. For instance, the mean risk perceptions for headaches were similar for verbal and numerical descriptors (46% vs. 41%) but the spread of risk estimates was much higher for verbal descriptors (standard deviation of 29% vs. 17%). While verbal descriptors might appear to perform adequately at the population level, these data imply that many individuals still will have over- or, just as importantly, under-appreciated their chance of developing side effects and adverse events [3,7,8].

Without a benchmark for descriptors such as “common” (i.e., common compared to what?), individuals must supply their own comparator and estimate risk in the context of their own beliefs, biases and experiences [12]. For example, someone who received the first dose of a COVID-19 vaccine and developed a fever may interpret “common” very differently than someone who did not. Prior studies have shown that in addition to personal factors, the nature of the data itself matters as well; for instance, side effects of different severity described with the same verbal probability term are often interpreted to have different likelihoods of occurring [13,14,15].

Of course, numerical descriptors are not a panacea. Guidelines for how to present such information—for example as percentages, natural frequencies, or graphical displays—are not always clear for all situations [12]. Numerical values may also convey a degree of certainty in information that is not supported by existing evidence. These dynamics may have combined with individuals’ past experiences or beliefs to explain the variability observed in the numerical descriptor arm between the estimates provided to participants and their self-reported risk perceptions. Future work should seek to capture and then address these potential shortcomings, which can be performed in several ways. One is to present data in the form of ranges instead of precise point estimates. Another is to provide contextual cues and graphical displays to place numerical data in the context of events that are likely more familiar to patients [12].

These steps are particularly worthwhile given the ubiquity of social media [16] and findings from our study. In particular, risk perceptions about myocarditis were greater among individuals reporting high social media influence. Though more work is needed to elucidate specific mechanisms behind this relationship, our findings nonetheless highlight the potential impact of social media on risk perceptions and the importance for various stakeholders to recognize this potential dynamic in determining formats for communicating risk probabilities [6,17].

Study limitations include the use of an online sample that may not represent all populations or capture changes in perceptions over time. However, while there may be differences between our sample and populations in either the US or other countries, the MTurk population has been used extensively for research in the social and health sciences [9,10,18,19]. Our findings are also highly salient to the broader pandemic situation given the prevalence and consistent use of verbal descriptors by public health and news organizations. Moreover, our findings are nonetheless valid given the randomized design and the goal of using randomization was to estimate the marginal effect of risk communication format between groups, rather than describing perceptions in a given population. Future work should elucidate changes in perceptions over time in different populations such as non-US populations and those with different levels of numeracy, graphical literacy and risk tolerance [12]. An additional limitation of this study is the overall lack of a validated survey instrument for the measurement of risk perceptions based on risk descriptors. However, the methodology employed in our study has been used extensively in the literature to assess risk perceptions in participants exposed to different risk communication formats [3,4,7,8,19]. We did not measure participants’ prior knowledge about vaccine adverse side effects or prior medical knowledge; however, the randomized design of this experiment should mitigate the impact of any bias introduced by these covariates on the marginal effect of risk communication strategies.

## 5. Conclusions

In conclusion, describing COVID-19 vaccine-related risks using verbal descriptors may lead to more inaccurate, variable and heightened perceptions of risk among US adults. Individuals reporting a high influence of social media may have heightened perceptions of some vaccine-related risks independent of risk communication format. Medical, public health and news media organizations can benefit from considering numerical risk estimates as an alternative to verbal descriptors in public communications to promote appropriate risk perceptions.

## Figures and Tables

**Table 1:** Participant characteristics.

Characteristic	Level	Numerical Probability	Verbal Probability
		*n* = 495	*n* = 444
Gender Identity, No. (%)			
	Male	271 (55%)	252 (57%)
	Female	219 (44%)	185 (42%)
	Trans Male/Transman	1 (0.2%)	0 (0%)
	Trans Female/Transwoman	0 (0%)	1 (0.2%)
	Genderqueer/Gender Non-conforming	0 (0%)	3 (0.7%)
	Prefer not to Say	4 (0.8%)	3 (0.7%)
Age ^a^, mean (SD), years		39 (11)	38 (12)
Identify with Hispanic Ethnicity, No. (%)			
	No	432 (87%)	385 (87%)
	Yes—Mexican, Mexican American, Chicano/Chicana)	25 (5.1%)	18 (4.1%)
	Yes—Puerto Rican	6 (1.2%)	1 (0.2%)
	Yes—Cuban	2 (0.4%)	2 (0.5%)
	Yes—Another Hispanic Origin	18 (3.6%)	25 (5.6%)
	Prefer not to Say	12 (2.4%)	13 (2.9%)
Racial Identity, No. (%)			
	American Indian/Alaska Native	4 (0.8%)	5 (1.1%)
	Asian Indian	15 (3.0%)	20 (4.5%)
	Black/African American	52 (11%)	42 (9.5%)
	Chinese	6 (1.2%)	8 (1.8%)
	Filipino	4 (0.8%)	0 (0%)
	Japanese	2 (0.4%)	2 (0.5%)
	Korean	2 (0.4%)	1 (0.2%)
	Multiple Identities	11 (2.2%)	14 (3.2%)
	Other Asian	3 (0.6%)	6 (1.4%)
	Other Pacific Islander	1 (0.2%)	0 (0%)
	Vietnamese	3 (0.6%)	7 (1.6%)
	White/Caucasian	379 (77%)	331 (75%)
	Prefer not to say	13 (2.6%)	8 (1.8%)
Employment Status, No. (%)			
	Employed Full Time	324 (65%)	274 (62%)
	Employed Part Time	48 (9.7%)	49 (11%)
	Unemployed (Looking for Work)	21 (4.2%)	24 (5.4%)
	Unemployed (Not Looking for Work)	19 (3.8%)	12 (2.7%)
	Retired	12 (2.4%)	15 (3.4%)
	Student	8 (1.6%)	9 (2.0%)
	Self-Employed	58 (12%)	58 (13%)
	Prefer Not to Say	5 (1.0%)	3 (0.7%)
Annual Household Income, No. (%)			
	<$25,000	71 (14%)	80 (18%)
	$25,000–$49,999	154 (31%)	111 (25%)
	$50,000–$74,999	121 (24%)	121 (27%)
	$75,000–$99,999	69 (14%)	68 (15%)
	$100,000–$124,999	32 (6.5%)	20 (4.5%)
	$125,000–$149,999	20 (4.0%)	17 (3.8%)
	>$150,000	19 (3.8%)	20 (4.5%)
	Prefer Not to Say	9 (1.8%)	7 (1.6%)
Education Level, No. (%)			
	Less than High School	4 (0.8%)	3 (0.7%)
	High School Diploma	64 (13%)	53 (12%)
	Some College, no degree	108 (22%)	111 (25%)
	Bachelor’s Degree	255 (52%)	221 (50%)
	Master’s Degree	48 (9.7%)	44 (9.9%)
	Professional Degree	7 (1.4%)	7 (1.6%)
	Doctorate Degree	4 (0.8%)	4 (0.9%)
	Prefer not to Say	5 (1.0%)	1 (0.2%)
Political Identity ^b^, mean (SD)		4.13, (1.86)	4.20, (1.88)
	Strongly Conservative	43 (8.7%)	36 (8.1%)
	Conservative	80 (16%)	73 (16%)
	Somewhat Conservative	59 (12%)	53 (12%)
	Neither conservative nor liberal	103 (21%)	83 (19%)
	Somewhat Liberal	67 (14%)	63 (14%)
	Liberal	91 (18%)	80 (18%)
	Strongly Liberal	45 (9.1%)	52 (12%)
	Prefer not to Say	7 (1.4%)	4 (0.9%)
Intentions to get next indicated dose of a COVID-19 vaccine, No. (%)			
	Unsure—Don’t need more time to decide	27 (5.5%)	18 (4.1%)
	Unsure—Need more time to decide	150 (30%)	126 (28%)
	Will Get Vaccine	198 (40%)	191 (43%)
	Won’t Get Vaccine	120 (24%)	109 (25%)
Social Media is Greatest Influence, No. (%)		102 (21%)	93 (21%)

SD = Standard Deviation, ^a^ Excludes *n* = 8 participants who indicated their age as > 90. ^b^ 7-point scale, higher values are more liberal.

**Table 2:** Results of multivariable linear regression models of the perceived risk of COVID-19 vaccine side effects.

		Headache		Fever		Fatigue		Myocarditis	
Covariate	Level	Beta (95% CI)	*p*-Value ^a^	Beta (95% CI)	*p*-Value ^a^	Beta (95% CI)	*p*-Value ^a^	Beta (95% CI)	*p*-Value ^a^
Probability Descriptor			<0.001		<0.001		0.002		<0.001
	Numerical Probability	Ref		Ref		Ref		Ref	
	Verbal Probability	5.0 (2.0, 8.1)		27 (23, 30)		4.9 (1.8, 8.0)		4.6 (2.1, 7.2)	
High Social Media Influence (Yes vs. No)		1.5 (−2.5, 5.5)	0.4	3.7 (−0.72, 8.0)	0.066	−2.9 (−6.8, 1.1)	0.14	6.7 (3.2, 10)	<0.001
Gender Identity ^b^									
	Female	Ref		Ref		Ref		Ref	
	Male	−4.8 (−7.8, −1.7)		−6.1 (−9.3, −2.9)		−5.6 (−8.7, −2.4)		−2.8 (−5.3, −0.21)	
	Non-Binary	−3 (−12, 6.5)		4.9 (−16, 26)		9.8 (−11, 30)		−3.2 (−14, 7.3)	
	Prefer not to Say	−29 (−45, −13)		−21 (−40, −2.4)		−13 (−33, 8.1)		−12 (−24, 0.22)	
Age (per 1 year)		0.14 (−0.01, 0.29)		0.26 (0.09, 0.43)		0.17 (0.01, 0.33)		0.26 (0.11, 0.42)	
Race ^b^									
	Non-White	Ref		Ref		Ref		Ref	
	Prefer not to Say	2.5 (−7.2, 12)		2.7 (−6.7, 12)		0.76 (−9.9, 11)		4.7 (−13, 3.6)	
	White/Caucasian	0.65 (−3.2, 4.5)		−1.5 (−5.5, 2.6)		1.8 (−2.2, 5.7)		−4.3 (−7.8, −0.92)	
Hispanic Ethnicity ^b^									
	Not Hispanic	Ref		Ref		Ref		Ref	
	Hispanic	3.2 (−2.1, 8.4)		6.4 (0.19, 13)		2.3 (−3.5, 8.1)		7 (1.8, 12)	
	Prefer not to Say	−2.6 (−14, 8.5)		−5.3 (−16, 5.2)		−11 (−22, −0.15)		5.2 (−4.3, 15)	
Employment Status ^b^									
	Employed	Ref		Ref		Ref		Ref	
	Prefer Not to Say	7.4 (−13, 28)		14 (−9.9, 38)		−2 (−21, 17)		3.3 (−9.2, 16)	
	Retired	−7.4 (−18, 3.2)		−5.3 (−16, 5.0)		−4.7 (−16, 6.2)		−0.36 (−12, 11)	
	Student	8.1 (−3.1, 19)		3.8 (−8.5, 16)		0.33 (−12, 13)		2.8 (−6.3, 12)	
	Unemployed	3.7 (−1.1, 8.6)		3.5 (−1.6, 8.5)		8.4 (3.2, 14)		−0.8 (−5.2, 3.6)	
Household Income ^b^									
	<$25,000	Ref		Ref		Ref		Ref	
	≥$25,000	−0.59 (−4.6, 3.4)		−0.62 (−5.0, 3.8)		−0.65 (−4.7, 3.4)		1.1 (−1.9, 4.2)	
	Prefer Not to Say	8.1 (−5.2, 21)		6.5 (−9.5, 22)		2.6 (−8.8, 14)		−2.3 (−10, 5.8)	
Education Level ^b^									
	Advanced Degree	Ref		Ref		Ref		Ref	
	Bachelor’s Degree	−0.92 (−5.6, 3.7)		−4.6 (−9.8, 0.61)		−4 (−8.7, 0.74)		−2 (−6.6, 2.7)	
	High School or Less	2.9 (−3.0, 8.7)		−2.7 (−9.1, 3.7)		−0.29 (−6.2, 5.6)		−2 (−7.4, 3.4)	
	Prefer not to Say	8.9 (−10, 28)		11 (−18, 41)		24 (10, 38)		10 (−7.0, 28)	
	Some College, no degree	2.1 (−3.1, 7.2)		−6.7 (−12, −1.1)		−1.9 (−7.1, 3.4)		−5.6 (−10, −1.0)	
Political Identity ^c^		−0.06 (−1.0, 0.86)		−0.64 (−1.6, 0.34)		0.85 (−0.09, 1.8)		−1.2 (−2.1, −0.31)	
Vaccine Hesitancy									
	Vaccine Accepting	Ref		Ref		Ref		Ref	
	Vaccine Deferring	5.6 (2.0, 9.2)		−2.0 (−5.8, 1.7)		7.4 (3.8, 11)		−4.9 (−7.9, −1.8)	
	Vaccine Hesitant	0.50 (−7.2, 8.3)		−6.8 (−14, 0.39)		−3.5 (−11, 4.4)		−4.5 (−9.2, 0.22)	
	Vaccine Rejecting	6.7 (2.5, 11)		0.52 (−3.9, 4.9)		7.9 (3.7, 12)		−1.5 (−5.5, 2.6)	

^a^ *p*-values are reported only for covariates related to pre-specified hypotheses. ^b^ Categories of some covariates with small cell values have been collapsed for the purposes of multivariate modeling when not related to the primary outcome of interest. ^c^ Higher is more Liberal on this scale of political identity. Model R^2^ values are 0.07 for headache, 0.27 for fever, 0.10 for fatigue and 0.11 for myocarditis.

## Data Availability

The data presented in this study are available on request from the corresponding author. The data are not publicly available due to need for privacy.

## Supplementary Materials

The following supporting information can be downloaded at: https://www.mdpi.com/article/10.3390/healthcare11030380/s1, Table S1: American Association for Public Opinion Reporting (AAPOR) reporting guidelines checklist.
