# Supplementary material for: Association between Risk Communication Format and Perceived Risk of Adverse Events after COVID-19 Vaccination among US Adults
Source: Healthcare (Basel). 2023 Jan 29;11(3):380. doi: 10.3390/healthcare11030380 (PMC9914684; doi:10.3390/healthcare11030380)
Supplement: Supplementary file 1 [file healthcare-11-00380-s001.zip › healthcare-2172587-supplementary.pdf]

Supplementary Material

Table S1: American Association for Public Opinion Reporting (AAPOR) reporting guidelines checklist

Checklist Item	Response
Survey Sponsor	University of Washington
Survey/Data Collection Supplier	Survey created on Qualtrics. Survey administered on Amazon’s Mechanical Turk platform via the CloudResearch Inc. interface
Population represented	Adults in the United States
Sample Size	2395 opened survey<br>284 indicated they were University of Washington employees and were removed<br>319 duplicate IP addresses removed<br>136 removed for completing < 99% of the survey<br>717 fully vaccinated/boosted removed<br>939 final sample
Mode of Data Collection	Internet survey
Type of sample	Non-probability
Start and end dates of Data Collection	1/10/2022 – 1/11/2022
Margin of sampling error for total sample	N/A
Are the data weighted?	No
Subgroup reporting	No
